# Event-based text mining for biology and functional genomics

**DOI:** 10.1093/bfgp/elu015

**Published:** 2014-06-06

**Authors:** Sophia Ananiadou, Paul Thompson, Raheel Nawaz, John McNaught, Douglas B. Kell

**Keywords:** text mining, event extraction, semantic annotation, semantic search

## Abstract

The assessment of genome function requires a mapping between genome-derived entities and biochemical reactions, and the biomedical literature represents a rich source of information about reactions between biological components. However, the increasingly rapid growth in the volume of literature provides both a challenge and an opportunity for researchers to isolate information about reactions of interest in a timely and efficient manner. In response, recent text mining research in the biology domain has been largely focused on the identification and extraction of ‘events’*,* i.e. categorised, structured representations of relationships between biochemical entities, from the literature. Functional genomics analyses necessarily encompass events as so defined. Automatic event extraction systems facilitate the development of sophisticated semantic search applications, allowing researchers to formulate structured queries over extracted events, so as to specify the exact types of reactions to be retrieved. This article provides an overview of recent research into event extraction. We cover annotated corpora on which systems are trained, systems that achieve state-of-the-art performance and details of the community shared tasks that have been instrumental in increasing the quality, coverage and scalability of recent systems. Finally, several concrete applications of event extraction are covered, together with emerging directions of research.

## BACKGROUND: THE LITERATURE DELUGE AND TEXT MINING

It is not news that science produces an enormous literature [1]—presently 23 million citations in MEDLINE® alone—and that computational means such as text mining (TM) are needed to extract meaningful knowledge from it. The biological literature in particular is largely focused on describing relationships between entities (e.g. genes, proteins and complexes), including how such entities interact and affect each other. Thus, biological TM research has focused extensively on the automatic recognition, categorisation [2] and normalisation of variant forms [3, 4] and mapping of these entities to unique identifiers in curated databases, e.g. UniProt [5]. This can facilitate entity-based searching of documents, which can be far more effective than simple keyword-based searches {see e.g. KLEIO (http://www.nactem.ac.uk/Kleio/) [6] and GeneView (http://bc3.informatik.hu-berlin.de) [7]}

As with systems biology [8], functional genomics is a prime candidate for TM (e.g. [9–12]). This is because one can automate the process of discovering relationships that hold between entities. A simple method of discovering ‘possible’ relationships is to find instances of sentences or abstracts in which groups or pairs of entities co-occur [13, 14]. This has been applied to the discovery of potentially unknown associations between different biomedical concepts [15]. However, such simple approaches, which do not consider the structure of the text, may generate incorrect hypotheses regarding relationships between entities. For example, only 30% of pairs of protein entities that occur in the same sentence actually represent an interaction [16]. More complex levels of textual processing, facilitated by the increasing availability of robust language processing tools tailored to biological text, such as deep syntactic parsers (e.g. [17]), can increase accuracy by limiting extracted relationships to those in which syntactic or semantic links hold between the entities.

Relationships between entities are widely referred to as ‘events’ [18, 19]*,* and their automatic recognition has become a major focus and rapidly maturing area of biomedical TM research. Increasingly ambitious community challenges [20–22] have been a major factor in the increasing sophistication of event extraction systems, both in terms of the complexity of the information extracted and the coverage of different biological subdomains. Moving beyond the simple identification of pairs of interacting proteins in restricted domains [23, 24], state-of-the art systems (e.g. [25, 26]) can recognise and categorise various types of events (positive/negative regulation, binding, etc.) and a range of different participants relating to the reaction, e.g. the cause, entities undergoing change, locations/sites and experimental conditions. Furthermore, emerging research is investigating how various textual and discourse contexts of events result in different ‘interpretations’, i.e. hypotheses, proven experimental observations, tentative analytical conclusions, well-known facts, etc. Although the exact nature of the discourse context can vary according to author characteristics (e.g. English biomedical scientific papers written by native speakers often show a higher incidence of uncertainty than those written by non-native speakers [27]), extraction systems that are able to recognise and capture various degrees and types of contextual details to produce semantically enriched events provide opportunities to develop more sophisticated applications.

**Figure 1:**
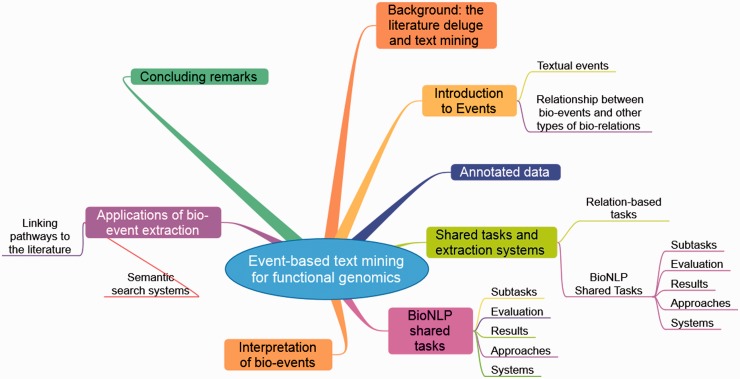
A ‘mind map’ summarising this Briefing. It should be read clockwise starting at 1 o’clock.

Event extraction systems can be used to develop applications (e.g. [28, 29]) that offer various benefits to the researchers, e.g. in facilitating more focused and relevant searches for information, in helping to locate literature-based evidence for reactions described in a pathway model or in detecting potential contradictions or inconsistencies in information reported in different articles. The purpose of this briefing, summarised as a Mind Map in Figure 1, is therefore to bring to readers’ attention how event-based TM approaches are providing considerable assistance to biological scientists struggling to cope with the literature deluge, and in particular, how they may be applied to the problems of functional genomics.

## INTRODUCTION TO EVENTS

### Textual events

A textual event may be described as an action, relation, process or state expressed in the text [30]. More specifically, it is a structured, semantic representation of a certain piece of information contained within the text, usually anchored to particular text fragments. These include the ‘trigger’, usually a verb or a noun that indicates the occurrence of the event, and ‘participants’, which may be assigned semantic roles according to their function. Typically, events and participating entities are assigned types/classes from taxonomies or ontologies. A bio-event is a textual event specialised for the biomedical domain, normally a ‘dynamic’ bio-relation in which at least one of the biological entities in the relationship is affected, with respect to its properties or its location, in the reported context [31].

**Figure 2:**
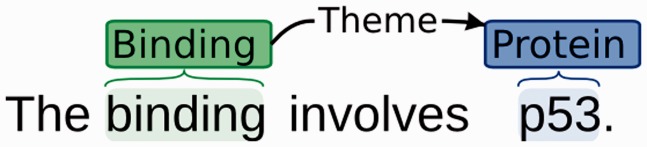
Simple bio-event example.

Figure 2 shows a very simple example of a bio-event. The trigger (*binding*) allows the semantic event type ‘Binding’ to be assigned. A single participant, *p53,* is identified as an entity of type ‘Protein’ and has been assigned the semantic role ‘Theme’, as it undergoes change as part of the event.

**Figure 3:**
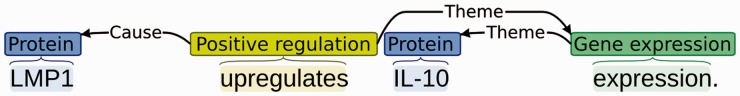
Sentence containing two events.

**Figure 4:**
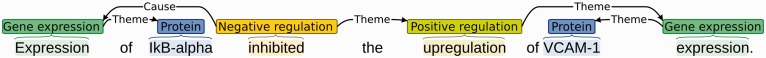
More complex sentence containing multiple events.

Figure 3 shows a more complex example, involving two events. First, the protein *IL-10* is identified as the Theme of the simple ‘Expression’ event. The verb ‘upregulates’ is the trigger for the second, complex event, which has been assigned the semantic event type ‘Positive regulation’. This event has two participants. The protein *LMP1* has been identified as the ‘Cause’ of the positive regulation event, while the Theme is the previously mentioned Expression event. Figure 4 shows a longer sentence, but illustrates how event structures can encode complex semantics and normalise over different means of linguistic expression (e.g. the two different Expression events).

### Relationship between bio-events and other types of bio-relations

The above general definition of a bio-event has been used as the basis for various annotation and extraction tasks [19, 31–34]. It can also encompass bio-relations, e.g. protein–protein interactions (PPIs) [35, 36], genotype–phenotype associations [37, 38], disease-gene associations [16, 39], drug–drug interactions [40], etc. Such relations can be considered to be a special type of bio-event with only two participants. For example, PPI extraction may determine that an (indirect) interaction holds between A and B in the sentence S1:

S1: A regulates the phosphorylation of B.

PPI extraction has been used to populate interaction databases, such as the Molecular INTeraction database (MINT) [41], which aims to collect information about experimentally verified molecular interactions (MIs). However, considering the semantics of S1 at a finer-grained level allows two separate events to be identified, with the triggers regulates and ‘phosphorylation’. This finer-grained analysis can be important, e.g. given that correlations between cellular components can be affected by both direct and indirect paths [42]. The more detailed results of bio-event extraction can be used to provide semantic enrichment of resources such as the Gene Wiki [10], a collection of more than 10 000 review articles, each describing a human gene, in which Gene Ontology (GO) [43] and Disease Ontology [44] terms have already been recognised automatically. Event extraction can also support the development and maintenance of more detailed and complex knowledge bases of biological processes and pathways (e.g. [45, 46]), which provide ready access to a wealth of information to support analyses and answer research questions.

## ANNOTATED DATA

Annotated collections of biomedical texts (known as corpora), in which domain experts have manually identified and marked up bio-events, provide direct and high-quality evidence of how events manifest themselves in texts. They are used to train event extraction systems, through the application of machine learning techniques to the annotated data, as well as acting as a ‘gold standard’ for evaluation [47].

Annotated corpora-identifying relations between pairs of concepts include the DDI corpus [48], consisting of 1025 textual documents (from the DrugBank database [49] and MEDLINE abstracts) annotated with 5028 drug–drug interactions, classified into four different types. The Fourth i2b2/VA shared-task corpus [50] contains 1354 clinical records (patient reports) in which eight types of relations that hold between-medical problems, treatments and tests have been annotated. The GeneReg corpus [51] identifies 1770 pairwise relations between genes and regulators in 314 MEDLINE abstracts that deal with the model organism *Escherichia coli*.Relations correspond to three classes in the gene regulation ontology (GRO) [52].

Regarding more complex event annotation corpora, BioInfer [32] captures events that can have more than two participants. Its 2662 bio-events, annotated in 1100 sentences from biomedical abstracts, are quite broad in scope, being assigned to one of the 60 different classes of the BioInfer relationship ontology. The GENIA event corpus [31] also uses a fairly complex ontology of 36 event types, based largely on a subset of classes from the GO. As one of the largest bio-event corpora, it consists of 1000 annotated abstracts concerning transcription factors in human blood cells, with 36 858 events. Participants include Location, Time and Experimental Context, in addition to Theme and Cause. Negation and speculation information is also annotated. The Gene Regulation event corpus [53] is more restricted in terms of domain, size and event types (240 MEDLINE abstracts relating to the *E. coli* and human species, with 3067 bio-events). However, its unique feature is its rich set of event arguments—13 different semantic role types are annotated.

The three BioNLP Shared Task (ST) competitions [19, 20, 54–56] have evaluated various event-based information extraction tasks, based around common sets of training and test data. They have contributed 11 event-annotated corpora, varying according to text type (full papers or abstracts), bio-medical subdomain and/or target application area. The STs have encouraged the development of increasingly practical and wide coverage event extraction systems (see next section). The multi-level event extraction corpus [57] also aims at improving coverage of event extraction systems, through its annotation of information pertaining to multiple levels of biological organisation, from the molecular to the whole organism.

## STs AND EXTRACTION SYSTEMS

STs bring together different research teams to focus on timely issues by providing standard datasets and a common evaluation framework [58]. They have played a significant role in advancing the state of the art in various types of biomedical TM systems [59, 60], including information retrieval (TREC Genomics track [61]) and named entity recognition {JNLPBA [62] and several BioCreAtIvE challenges since 2003 (http://www.biocreative.org/)}.

### Relation-based tasks

Challenges focusing on relations between pairs of entities have included the language learning in logic (LLL) challenge [22], concerned with identifying ‘genic’ interactions in MEDLINE abstracts. Machine learning-based methods representing training examples as sequences and the use of extended lists of words denoting interactions were found to be advantageous in this context. The drug–drug interaction (DDI) challenges task [63, 64] focused on the detection and/or four-way characterisation of interactions between pairs of drugs in texts from DrugBank [49] and MEDLINE abstracts. Support vector machines (SVMs) [65, 66] were used by many participating teams, with non-linear kernel-based methods demonstrating clear advantages over linear SVMs. In the fourth i2b2/VA Shared-Task [50], which was based around the aforementioned corpus involving relations between problems, treatments and tests, systems using SVMs were once again found to be the most successful. The highest F-scores achieved in the above challenges ranged from ∼42–74%, with quality affected by factors such as text type (academic abstracts versus less formal text), training data size (from 271 training examples for LLL to ∼5000 for i2b2/VA) and task complexity (e.g. whether relations had to be classified). (F-measure (yielding an F-score) is standardly used to report performance of TM systems. It considers both precision (number of correct results divided by overall number of results) and recall (number of correct results divided by the number of results known to be correct), when applied to a test sample and results compared with a gold standard annotation of that sample. Commonly, the balanced F_1_-score (harmonic mean) is reported.)

The BioCreative challenges [60, 67, 68] have addressed a number of biological TM tasks, such as biomedical named entity recognition and normalisation, and PPI extraction (BioCreative II [67] and II.5 [69]). In contrast to other STs, the gold-standard interactions were not text-bound, but rather consisted of a normalised list of entity pairs for each full-text article. A range of methods was used to extract and normalise these pairs, including machine-learned sentence classifiers, detection of interaction-relevant verbs, keywords or word patterns, rules, use of syntactic parser output and the relative position of relevant sentences within the full-text article. However, the best results achieved (29% and 22% F-score for BioCreative II and II.5, respectively) illustrate the increased complexity when gold standard text-bound training data are not available.

### BioNLP STs

The three BioNLP STs [19, 20, 34] have focused on a number of generally more complex event and relation extraction problems than those introduced above, including the recognition and classification of event triggers, multiple participants and information about event interpretation (e.g. negation and speculation). Different ST tasks have varied in terms of text type, biological subdomain and event types covered, thus helping to encourage the development of increasingly robust, sophisticated and wide coverage systems. Table 1 provides an overview of the tasks and results for each task. The 2013 BioNLP ST mapped each task to an overarching objective: i.e. to apply different tasks to construct a knowledge base for systems biology needs [20]. The GENIA event extraction (GE) task targeted knowledge base construction, pathway curation (PC) aimed at supporting development of pathway models, Cancer Genetics (CG) focused on the molecular mechanism of cancer, gene regulation network in bacteria (GRN) was concerned with regulation networks and corpus annotation with GRO dealt with ontology population.

**Table 1:** BioNLP shared task details

Task	Subtask	Participants	Text type	GENIA model	Event types	Best system	Approach	Accuracy
BioNLP’09	GE [19]	24	A	Y	9	TEES [70]	SVM + rules pipeline	54.89
BioNLP’11	GE [54]	13	F	Y	9	UMASS [71]	Joint inference	53.14
			A			FAUST [72]	Stacking: UMASS + Stanford pipeline (MaxEnt + MSTParser)	57.46
			A + F			FAUST [72]	Stacking (as above)	56.06
	EPI [55]	7	A	Y	14	TEES 2.0 [73]	SVM pipeline	53.33
	ID [55]	7	F	Y	10	FAUST [72]	Stacking (as above)	57.57
	BI [56]	1	A	N	10	TEES 2.0 [73]	SVM pipeline	77.0
	BB [56]	3	W	N	2	Bibliome [74]	Co-occurrence of arguments and triggers	45.0
BioNLP’13	GE [75]	10	F	Y	13	EVEX [76]	SVM pipeline	50.97
						TEES 2.1 [26]	SVM pipeline	50.74
						BioSEM [77]	Rule pipeline	50.68
	CG [78]	6	A	Y	40	TEES 2.1 [26]	SVM pipeline	55.41
	PC [79]	2	A	Y	23	EventMine [80]	SVM pipeline	52.84
	GRO [81] (Relation)	2	A	N	8	TEES 2.1 [26]	SVM pipeline	63.00
	GRN [82]	5	A	N	12	U. Ljubliana [83]	Linear chain CRF + rules	0.73 (SER)
	BB [84]	5	W	N	2	TEES 2.1 [26]	SVM pipeline	42.00

GE = GENIA event; EPI = epigenetics and post-translational modifications; ID = infectious diseases; GI = gene interaction; BB = bacteria biotope; CG = cancer genetics; PC = pathway curation; GRO = gene regulation ontology; GRN = gene regulation network. For text type, A = abstracts; F = full papers and W = web pages. The ‘GENIA model’ column indicates whether events were based on the GENIA event model. The accuracies of the reported systems correspond to F-scores, apart from the GRN task, which is reported in terms of slot error rate (SER) (the lower, the better, in the range 0–1).

#### Tasks

Each ST has included a GE (GENIA Event) task, using the same textual subdomain (i.e. molecular biology) as the original GENIA event corpus, and a subset of the original event types. The BioNLP’09 task [85] was largely based around a simplified subset of the original GENIA event corpus [31], using only 9 of the original 36 event types, to make the event extraction problem more tractable. Subsequent GE tasks have added complexity by supplementing abstracts with full papers (BioNLP’11) [54], or by using an exclusively full-paper corpus, annotated with an extended range of event types (BioNLP’13) [75]. Several other tasks in the BioNLP’11 and BioNLP’13 STs have used a comparable event annotation model to GE, i.e. the tasks epigenetics and post-translational modifications (EPI), infectious diseases (ID) [55] (BioNLP’11), CG [78] and PC [79] (BioNLP’13). Each of these tasks defined a set of event types relevant to the corresponding subdomain and/or target task. Some other tasks used custom (non-GENIA) representations for events or relations.

#### Evaluation

GE tasks were evaluated by splitting the problem as follows:-subtask 1—locating bio-event triggers, assigning event types and identifying core participants (i.e. Theme and Cause); subtask 2—identifying additional participants, including locative information; subtask 3—identifying negation and speculation. As only subtask 1 was obligatory and participation in subtasks 2 and 3 was much smaller, results for the GE subtasks reported in Table 1 concern subtask 1. In contrast, for the EPI, ID, CG and PC tasks, the standard means of evaluation encompassed full event extraction in one, including the recognition of additional arguments, negation and speculation.

#### Results

The best performing systems extracting GENIA-style events have achieved accuracy levels between 50 and 57% F-score, depending on task and domain. This is considered encouraging, given that the quality of systems has consistently improved in successive STs (comparing results on the GE abstract dataset in 2009 and 2011), but also because the output quality can be fairly stably maintained when variations occur in text type, bio-medical subdomain and event types. Particularly notable are the PC and CG tasks, because the results are comparable with those achieved in earlier GE tasks, despite the considerably increased complexity of event types and the more demanding full event extraction criteria. For example, the top performing system in the CG task achieved a recall of 48.76% and a precision of 64.17%, although the performance of the second best system was more balanced, i.e. 48.83% recall and 55.82% precision. Regarding tasks with custom event/relation representations, some simpler tasks produced higher accuracies than the GENIA-based tasks, e.g. the bacteria interaction (BI) task [56] of BioNLP’11, which provided entities, triggers and syntactic parses as gold standard data, and the GRO relation extraction task of BioNLP’13, which identifies only pairwise relations [81]. The lower scores achieved in the bacteria biotope tasks of BioNLP’11 [56] and BioNLP’13 [84] (45% recall/45% precision and 28% recall/82% precision, respectively) reflect the complexity of the task, requiring the resolution of many instances of co-reference (i.e. cases where two or more expressions in a text refer to the same entity), and dealing with the occurrence of many inter-sentential events. Overall, the performance of event extraction systems depends on the domain, the nature of the task and the types of entities involved. For example, it was demonstrated in [57] that events involving anatomical entities are more reliably extracted than molecular level events, with performance levels for the former types of events reaching 80.91% precision, 72.05% recall and 76.22% F-score, despite the fact that the annotation corpus contained a larger number of molecular level events.

#### Approaches

Pipeline-based machine-learning approaches have performed consistently well on many different tasks. Such systems generally implement separate modules to perform the following: (a) identify event triggers, (b) detect separate arguments of these triggers and (c) construct complex event structures from the trigger-argument pairs. As seen elsewhere with some relation-based extraction tasks, SVMs appear to be the most effective learning technique across most BioNLP ST tasks. However, other approaches have demonstrated competitive performance for certain tasks, e.g. a rule-based approach (BioSEM [77]), and a joint model with minimal domain adaptation (UMass system [71]). The latter was particularly effective when combined with information from Stanford’s parser-based model [86] in the stacking-based FAUST system [72]. For the non-GENIA event based extraction tasks, custom solutions can work well (e.g. [74]).

#### Systems

EventMine [87] is pipeline-based event extraction system that has been applied to several biomedical event extraction tasks. Its machine learning approach, based on SVMs, facilitates ease of portability to new tasks, through training on different corpora. The robustness of the system has also been illustrated through its application to the entire PubMed abstract collection, the results of which are used to facilitate semantic event-based searching in the MEDIE search system [28] (see the section ‘Applications of Bio-Event Extraction’ for further details). It achieved first and second place in the PC and CG tasks of the BioNLP’13 ST, respectively, with the highest recall for both tasks [80]. EventMine achieved the best results on BioNLP’09 ST data (although it did not participate in the challenge), and obtained significantly better results for complex events (i.e. those that include other events as participants) than those systems originally participating in the challenge. A subsequent version of EventMine incorporated a new co-reference detection system (important, given the high occurrence of co-references in full papers [54]) and domain adaptation techniques [25], which allow features from multiple annotated corpora to be incorporated into the trained model. The updated system achieved further improved results on the BioNLP’09 ST data, and was also able to outperform all original participants in the BioNLP’11 GE and ID tasks (with F-scores 58.0 and 57.6%, respectively), both of which involved the extraction of events from full papers. A further improvement to EventMine allows the creation of a single event extraction system with broad semantic coverage, through training on multiple corpora with partial semantic annotation overlap [88]. A final enhancement to EventMine, making it unique in comparison to related systems, allows extracted events to be enriched with extended information about their interpretation according to textual and discourse context [89] (see the section ‘Interpretation of Bio-Events’).

The Turku event extraction system (TEES) [70] has participated in the majority of tasks of each of the three STs, and achieved the best performance in the GE tasks of BioNLP’09 and BioNLP’13, the EPI and BI tasks of BioNLP’11 and the CG, GRO relation and the BB tasks of BioNLP-13. Increased generalisability of TEES has been achieved through evolution from a partial rule based to a completely SVM-based pipeline [73], and incorporation of automated annotation scheme learning from training corpora, to allow adaptation to new tasks without human effort [90]. The system has been used to extract more than 19 million events from 18 million PubMed abstracts [91] and also to create the EVEX database [91–94], containing more than 40 million events from both abstracts and full papers. Information in EVEX was used to re-rank output from TEES in the BioNLP’13 GE subtask, resulting in a modest improvement in performance over the use of TEES alone [76].

FAUST [72] is distinct from TEES and EventMine in its usage of a stacking technique (a type of ensemble learning technique, i.e. a way of combining models rather than using a single model). Two previously competing models, from the University of Massachusetts and Stanford University, respectively, were configured such that the UMass model used the output (modulo re-ranking) of the parser-based model of Stanford as additional features. The combination of the differing features used in the two models resulted in FAUST achieving the best performance in three of the four tasks in which it participated in the BioNLP’11 ST. An interesting additional result was that novel events proposed by the stacking technique (i.e. where neither individual-base model had recognised such events) had very low precision, and that removal of such events from the output improved performance.

## INTERPRETATION OF BIO-EVENTS

Most current event extraction systems are trained on BioNLP ST corpora, which contain only limited annotations relating to event interpretation, e.g. negation and speculation. The binary distinction between speculated and non-speculated events made in these corpora is over-simplistic, as speculation can occur, or be expressed, in multiple degrees. In addition, further interpretative information about events can be distinguished. For example, an event may be presented as the subject of an investigation, a known fact, experimental observation or the outcome of analysing experimental results. Furthermore, events may represent knowledge cited from a previously published paper, or constitute part of the new knowledge contribution in the paper under consideration. Indeed, the nature of evidence underpinning scientific claims or belief is an important part of the GO annotations [43] and of modern means of annotating systems biology models [95–97].

Depending on the nature and criticality of the task being undertaken, some or all of the above distinctions may be important when searching for instances of events. Tasks such as building and updating models of biological pathways and curation of biological databases [98] require the identification of new and reliable experimental knowledge. Meanwhile, checking for inconsistencies or contradictions in the literature could be detected by examining events with identical participants but different interpretations.

Various efforts have assigned interpretative information at the sentence or clause level in academic articles (e.g. [99–102]). However, as a particular sentence may contain multiple events, each with their own interpretation, a new model has been proposed to identify distinct aspects of discourse interpretation (or ‘meta-knowledge’ dimensions) at the event level [103]. The model contains five dimensions, each of which has a fixed set of values. The dimensions are: ‘Knowledge Type (KT)’ (general type of information expressed by the event), ‘Manner’ (rate or intensity level of the described reaction), ‘Certainty Level (CL)’ expressed towards the event, the ‘Source (Src)’ of the information expressed by the event (new information in the paper under consideration, or information previously reported elsewhere and ‘Polarity’ (i.e. whether the event is negated).

**Figure 5:**
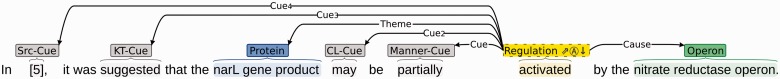
Annotated meta-knowledge example. The core elements of the event (i.e. the trigger for the *Regulation* event, and its *Theme* and *Cause* participants) have been enriched through the identification of cues that are relevant to various dimensions interpretation of the event, according to the meta-knowledge model.

As an example of how the model applies to an event within a specific discourse context, consider the sentence shown in Figure 5. There is a single event of type Regulation (triggered by the verb ‘activate’), which has two participants. The Cause of the event is ‘narL gene product’ and the Theme is ‘nitrate reductase operon’. The textual context of the event provides several important pieces of information about its interpretation, each of which conveyed by the presence of a specific cue word.

- The presence of the citation [5] indicates that the event does not report novel information but rather concerns details from a previous publication. Thus, the citation acts as a cue to denote that the value of the ‘Src’ dimension should be set to ‘Other’.
- The word ‘suggested’ denotes that within the previous publication, the event was not stated as definite, but rather was outcome of an analysis. This is a cue for a ‘KT’ value of ‘Analysis’.
- The confidence in the validity of the analysis is rather tentative, as denoted by the word ‘may’. Thus, the ‘CL’ value is ‘L1’ (the lowest of the three possible levels).
- The word ‘partially’ shows that the level/intensity of the proposed interaction is lower than would be expected by default. According to the model, the value of ‘Manner’ dimension is set as ‘Low’.

The meta-knowledge model has been applied manually to enrich the GENIA event corpus [104]. Event level meta-knowledge has been shown to complement more coarse-grained annotation schemes [105] and some significant differences between the distributions of meta-knowledge in full papers and abstracts have been revealed [106]. Experiments have demonstrated the feasibility of predicting values for Manner and ‘Polarity’ dimensions automatically [107, 108], while the enhanced EventMine can fully automatically extract events with such meta-knowledge information attached [89].

## APPLICATIONS OF BIO-EVENT EXTRACTION

Automatic extraction of bio-events has a broad range of applications [58], including support for the creation and annotation of pathways [109, 110], automatic population/enrichment of databases [111] and semantic search systems.

### Semantic search systems

**Figure 6:**
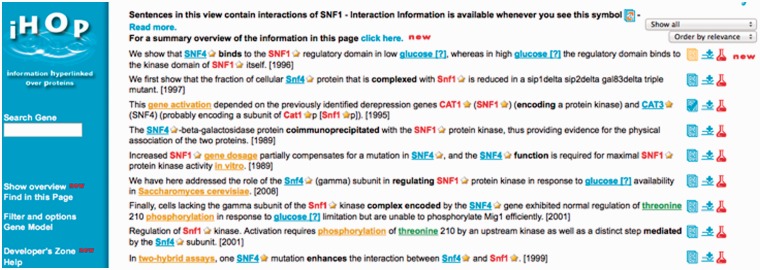
iHop search interface, showing results retrieved by search for *SNF1.* Additional entities, MeSH terms, interactions and words are highlighted. (A colour version of this figure is available online at: http://bfg.oxfordjournals.org)

Semantic search systems allow much more precise and focused retrieval and extraction than do the traditional keyword-based systems [112]. Earlier systems aimed to increase the number of hits retrieved by a user’s query, through automatic query expansion with synonyms or variants of query terms. Automatic identification of other terms and/or interaction-indicating verbs in the same sentence or abstract can allow identification of potential events or associations involving search terms. iHOP (http://www.ihop-net.org) [23, 113] highlights additional terms and verbs in sentences retrieved by searching for a gene (see Figure 6), whereas FACTA+ (http://www.nactem.ac.uk/facta/) [15] calculates and visualises strengths of association between a search term and other important concepts (e.g. genes, diseases and chemical compounds), by finding abstract-level co-occurrences over the whole of the MEDLINE abstract database. FACTA+ queries can be refined through specification that event(s) of a particular type should be present in the abstracts retrieved. For example, the query ‘ERK2 GENIA:Positive_regulation’ will retrieve abstracts containing both the term ‘ERK2’ and an event of type ‘Positive regulation’.

**Figure 7:**
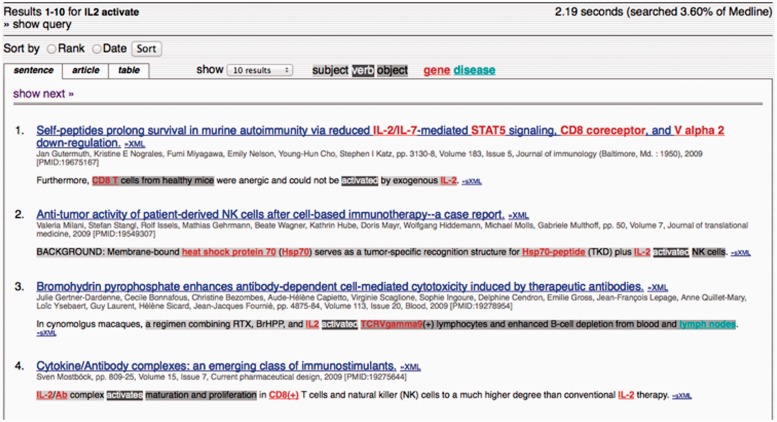
MEDIE search results. Relevant sentences from retrieved abstracts are shown, with separate colours for the subject, object and verb. (A colour version of this figure is available online at: http://bfg.oxfordjournals.org)

MEDIE [28] allows more precise, structured searching, through the application of a deep syntactic analyser tuned to the biomedical domain [114], combined with an event expression recogniser and a named entity recogniser [115]. Structured queries take the form of ‘<subject, verb, object>’ to specify an event, where ‘subject’ and ‘object’ refer to grammatical relations with the verb. Such relations often hold between the primary participants of events, and are the basis of the well-known Resource Description Framework (RDF) triple scheme [116]. Query results are shown in Figure 7. The subject, verb and object of the relation are highlighted separately in the relevant snippets of texts within the retrieved articles.

A recently released enhanced prototype of MEDIE (http://www.nactem.ac.uk/medie/ev-search.html) allows search criteria to be specified based on the GENIA event model, facilitated by applying EventMine to the PubMed abstract collection. This allows search criteria to abstract further from the surface structure of the text.

**Figure 8:**
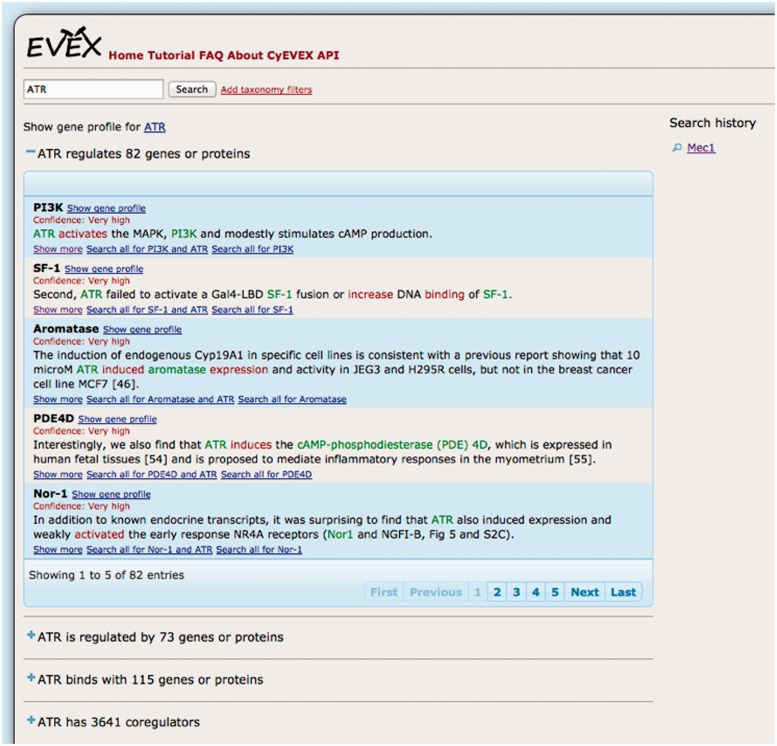
Interface to EVEX database, showing results after searching for the gene ATR.

Another event-based system offers a user interface over the EVEX database [94], allowing search based on the 40 million bio-molecular events extracted from 21.9 million PubMed abstracts and 460 000 PubMed Central open access full-text articles. Selecting a particular gene causes the event types in which it participates to be identified. In Figure 8, the events displayed involve the gene *ATR.* The statement ‘*ATR* regulates 82 genes or proteins’ denotes that *ATR* has been identified as the Cause of regulation events, in which 82 unique genes or proteins have been identified as the Theme. An example of an event involving each of these genes/proteins is displayed. For each gene/protein, links allow the user to further ‘drill down’ to information of interest, e.g. to find further examples of the given event type with a specific Cause and Theme, or to discover further event types involving a specific pair of genes/proteins. The events displayed in Figure 8 provide further evidence of how discourse contexts are important in distinguishing between different event interpretations (as explained in the section ‘Interpretation of Bio-Events’ above), and thus that such search systems could benefit from taking this information into account. For example, in the first row, which describes an interaction between *ATR* and *Nor1*, the word ‘find’ denotes that the event is stated based on experimental observations, while the word ‘weakly’ denotes that intensity of the regulation is very low.

EvidenceFinder (http://labs.europepmc.org/evf) has been developed to allow event-based filtering of search results and efficient location of information within >2.6 million articles from PubMed and PubMed Central contained within the Europe PubMed Central database. A recently released update of this interface (http://www.nactem.ac.uk/EvidenceFinderAnatomyMK/) is tailored to searching for anatomical entities, and enhances the functionality of other semantic search interfaces through the inclusion of extended filtering facilities, based on meta-knowledge extracted about the event, according to the model introduced above.

**Figure 9:**
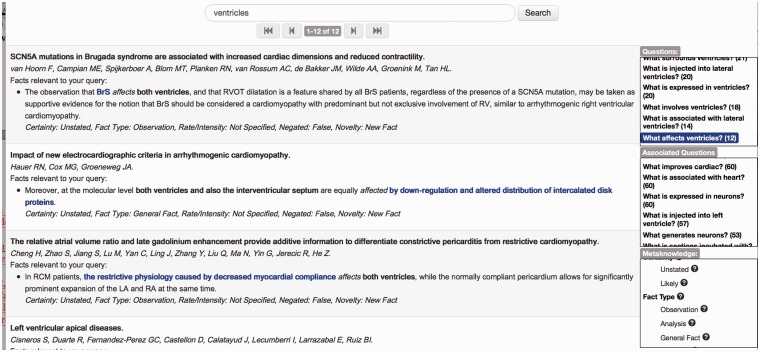
EvidenceFinder interface for anatomical entities.

For any given anatomical entity, e.g. ‘ventricles’, there can be many different types of events that mention the entity. Given such a search term, EvidenceFinder helps the user to filter the search results by generating a list of questions [117] that illustrate the most frequent types of events in which the search entity is involved in the Europe PubMed Central document collection (see the top right-hand box in Figure 9). In Figure 9, the question *What affects ventricles?* has been selected, and text snippets containing events that answer this question are shown on the left-hand side of the screen.

Events are extracted via a number of domain-specific tools and resources, namely the Enju Parser adapted to the biomedical domain [114], a named entity recogniser [118] and information about patterns of verb behaviour in biomedical texts, which is obtained from a large-scale domain-specific lexical resource, the BioLexicon [119]. This resource includes, amongst other information, details about the grammatical and semantic behaviour of verbs.

The event extraction process used in EvidenceFinder additionally includes the assignment of meta-knowledge information to events. For the first result in the list in Figure 8, the ‘Fact Type’ is set to ‘Observation’, because the textual context reveals that the event is stated based on experimental findings. In contrast, the second result states generally accepted information (probably as background to new research being carried out), and hence the Fact Type is set to the ‘General Fact’. The ‘Meta-knowledge’ box allows one or more specific values to be selected to refine the search results according to the varying event interpretations.

### Linking pathways to the literature

Biochemical signalling and metabolic pathways are becoming increasingly important for biomedical research, because they represent collective interpretations of facts scattered throughout the literature [96, 120–125]. The compilation, curation, annotation and maintenance of pathway models require substantial human effort, including reading previously published papers, monitoring the appearance of new ones and interpreting their results [126]. Furthermore, because different interpretations of the same set of facts are possible, not to say widespread (see e.g. [127, 128])), researchers often want—and intellectually ought—to read the original papers from which, e.g. a pathway is constructed [121, 129]. TM tools can be valuable, not only to support the maintenance of pathway models [130], but also to provide direct links from pathways to the supporting evidence in literature [95].

**Figure 10:**
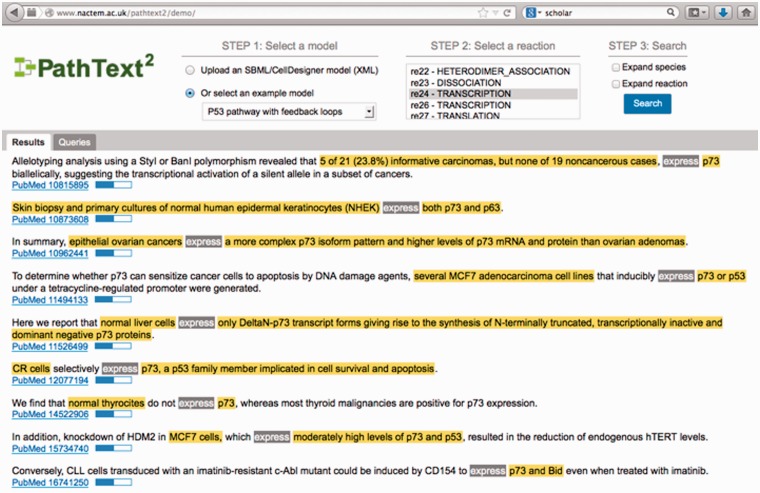
PathText 2 Interface.

PathText 2 (http://www.nactem.ac.uk/pathtext2/demo/) [109] is an integrated search system that links biological pathways with supporting knowledge in the literature. It reads formal pathway models (represented in the Systems Biology Markup Language (SBML) [131] with CellDesigner [132]) and converts them into queries that are submitted to three semantic search systems operating over MEDLINE, i.e. KLEIO [6], which improves and expands on standard literature querying with semantic categories and facetted search, FACTA+ and MEDIE (both the original and GENIA event-based versions). The average hit ratio of each system (i.e. the fraction of queries generated by PathText 2 that retrieve a given document) is considered when ranking the documents. The GENIA event-based version of MEDIE was found to achieve the highest hit ratio, demonstrating the superiority of this search method. Accordingly, documents retrieved by this method are ranked first by the system. Figure 10 shows the PathText 2 interface. An SBML model is selected or uploaded, and a reaction is chosen. Textual evidence for the queried reaction in retrieved documents is displayed in the interface, along with a confidence score.

## CONCLUDING REMARKS

In recent years, the sophistication of automated methods to recognise relationships between entities in biomedical texts has increased considerably, moving from calculation of simple co-occurrence to the detection of pairwise relations between interacting proteins and to the extraction of sophisticated event structures involving multiple, categorised participants.

Complex event extraction systems can benefit researchers in a number of ways. Given the rapidly expanding volume of literature, semantic search systems allow far more efficient retrieval of relevant information than traditional keyword-based methods. Event extraction can also assist with tasks such as the semi-automatic curation of biomedical databases and ontologies and the linking of biological pathways with supporting evidence from the literature.

Community STs and associated event-annotated corpora have ensured that event extraction has developed into, and remains, an active research area. Systems dealing only with abstracts in restricted subdomains have given way to more flexible and adaptable systems, which, by incorporating techniques such as co-reference resolution or domain adaptation methods, can operate with comparable accuracy on different text types and domains with minimal, or even completely automatic, adaptation. Recent development of an event-based meta-knowledge model is opening up new research directions, including increasing the search possibilities of event-based search systems.

State-of-the-art event extraction technology is now accurate and robust enough to support the development of useful applications, as illustrated by our descriptions of several real-world applications. Developments in deep neural network learning (e.g., [133–135]) seem destined to improve this yet further. Application-oriented usage of event extraction has further been stimulated by the BioNLP 2013 ST, with the theme of *knowledge base construction*. However, further such initiatives are needed, in order that future efforts to improve event extraction technology are balanced by efforts to exploit it more extensively in user-oriented applications, thus ensuring that the full practical potential of event extraction technology is realised and appreciated by the biomedical community.

As the community focuses on improving the domain independence of annotations and methods, complex event extraction at large scale will become a core technology in the world of Big Data and Linked Open Data. Existing biomedical ontologies, databases and other resources provide the semantics to drive the TM systems. In turn, the output of the systems is used to further enrich the resources in a bootstrapping manner. This synergy between TM and enriched Linked Open Data is one of the cornerstones of the informatics infrastructure needed to support biomedicine. These efforts will support existing initiatives such as ELIXIR (http://www.elixir-europe.org) and BioCreaTiVe in facilitating the curation of large-scale biological databases and ontologies, together with the aggregation of workflows and services. As data floods entail further publications, the manual curation and update of numerous databases, using information from the literature, within a realistic timeframe, is a sine qua non. However, the integration of high-quality information of a complex nature, such as events extracted automatically from the literature, into bioinformatics platforms, will allow scientists to process and better comprehend the amount of data at their disposal. Sectors such as pharmaceuticals, biotechnology and biocatalysis rely on high quality, comprehensive, accurate and timely information, which TM can provide. Big Data is here, and TM is essential to allow us to use and make sense of it to support science.

**Key points**

- The enormous volume of biology literature demands computational methods to allow pertinent information to be found and analysed efficiently.
- TM facilitates the extraction from documents of semantic information such as entities (proteins, genes, etc.) and events (binding, regulation, etc.) in which the entities participate.
- Recent community STs have encouraged and led to the development of increasingly accurate and wide coverage event extraction systems.
- Event extraction systems are now sufficiently accurate to support the development of various user-oriented applications, including sophisticated semantic search, and means for linking biochemical pathways to evidence in the literature.
- Emerging research into the automatic assignment of interpretative information (meta-knowledge) to events can increase the power of event-based applications.
